# Informatics and Computational Methods in Natural Product Drug Discovery: A Review and Perspectives

**DOI:** 10.3389/fgene.2019.00368

**Published:** 2019-04-30

**Authors:** Joseph D. Romano, Nicholas P. Tatonetti

**Affiliations:** ^1^Department of Biomedical Informatics, Columbia University, New York, NY, United States; ^2^Department of Systems Biology, Columbia University, New York, NY, United States; ^3^Department of Medicine, Columbia University, New York, NY, United States; ^4^Data Science Institute, Columbia University, New York, NY, United States

**Keywords:** drug discovery, methods, cheminformatics, bioinformatics, ontologies, translation, natural products

## Abstract

The discovery of new pharmaceutical drugs is one of the preeminent tasks—scientifically, economically, and socially—in biomedical research. Advances in informatics and computational biology have increased productivity at many stages of the drug discovery pipeline. Nevertheless, drug discovery has slowed, largely due to the reliance on small molecules as the primary source of novel hypotheses. Natural products (such as plant metabolites, animal toxins, and immunological components) comprise a vast and diverse source of bioactive compounds, some of which are supported by thousands of years of traditional medicine, and are largely disjoint from the set of small molecules used commonly for discovery. However, natural products possess unique characteristics that distinguish them from traditional small molecule drug candidates, requiring new methods and approaches for assessing their therapeutic potential. In this review, we investigate a number of state-of-the-art techniques in bioinformatics, cheminformatics, and knowledge engineering for data-driven drug discovery from natural products. We focus on methods that aim to bridge the gap between traditional small-molecule drug candidates and different classes of natural products. We also explore the current informatics knowledge gaps and other barriers that need to be overcome to fully leverage these compounds for drug discovery. Finally, we conclude with a “road map” of research priorities that seeks to realize this goal.

## 1. Introduction

Drug discovery is the process by which new pharmaceutical drugs are identified, and along with drug development (validating, testing, and marketing a new drug), it comprises one of the most substantial activities in pharmaceutical science. A 2018 analysis showed that roughly 20% of the US National Institutes of Health (NIH) budget for the years 2010–2016 funded the discovery and development of 210 new molecular entities (Cleary et al., 2018). Since the advent of modern medical science, most systematic drug discovery has focused on small molecule candidates—for example, over 86% of the drugs (both approved and experimental) in the DrugBank database are comprised of small molecules (Wishart et al., 2017). This is due to many reasons, including relative ease of synthesis, generally high chemical stability, and more straightforward characterization of reactivity (Drews, 2000). The pervasiveness of small molecules in drug discovery is even reflected in Lipinski's “rule of five,” which defines a set of common best-practice guidelines for filtering potential orally-active drug candidates: “Good” compounds should have a molecular mass <500, no more than five hydrogen bond donors, and no more than 10 hydrogen bond acceptors, among other principles (Lipinski, 2004).

In recent decades, the ubiquity of computers and computational methods in science has extended to drug discovery (Sliwoski et al., 2014). Cheminformatics, for example, is the application of computer science to understanding and characterizing molecular attributes and chemical behavior of specific compounds. These methods have generated massive libraries of small molecules to screen against specific therapeutic processes (Blaney and Martin, 1997). Once candidates are identified, other cheminformatics methods can be used to generate libraries of compounds structurally and chemically similar to the identified “hits,” in order to optimize stability, toxicity, and kinetics. Complementarily, bioinformatics techniques can be used to discover how candidate drugs cause therapeutic activity within the human body, which can include predicting interactions between drugs and proteins, analysis of impact on biological pathways and functions, and elucidating genomic variants that can alter drug response (Drews, 2000).

Despite these technological advances in drug discovery, the approval of new therapeutic drugs has slowed considerably in recent years. For example, between 1996 and 2007, the number of new molecular entities approved by the US FDA has fallen from 53 to 17 per year—the same rate as over 50 years ago (FitzGerald, 2008; Munos, 2009). This seems to be due to many factors, including the following:

1. The “lowest hanging fruits” in terms of small molecule drug candidates have been extensively investigated, and computational challenges hinder extension of traditional methods to more complex structures. Researchers refer to “rediscovering the sweet spot” in the discovery process (Brown and Superti-Furga, 2003), and have devoted a great amount of effort to producing new, targeted screening libraries that leverage anticipated characteristics of lead compounds (Welsch et al., 2010; Cheng et al., 2012).
2. Many remaining diseases of top clinical priority have highly complex etiologies, and are accordingly difficult to associate with potential drug targets (Ramsay et al., 2018).
3. Model organisms may not provide adequate templates for testing treatments of more complex diseases, due to inter-species variations that are crucial to therapeutic action (Hunter, 2008; Ehret et al., 2017).

A natural way to address the first two challenges is to focus on new classes of potential drugs outside of small molecules. Natural products (NPs) may serve this need by returning to the sources of therapeutic compounds that have treated illness for thousands of years (Dias et al., 2012). Although rigorous pharmaceutical science is young in comparison to the historical use of NP drugs, many cutting-edge advances have emerged with the promise of “modernizing” this field (Harvey, 2008). Along with a renewed interest for NP drugs within the biomedical research community, this has already resulted in substantial developments in the pharmaceutical industry—a comprehensive enumeration by Newman and Cragg shows that 41% (646/1562) of all new drug approvals between 1981 and 2014 are NPs or derived from NPs (Newman and Cragg, 2016). Several recent reviews provide excellent summaries of NP drugs and the broad spectrum of techniques that have been used both for their identification and characterization (Katz and Baltz, 2016; Rodrigues et al., 2016), particularly from the perspective of bench research techniques and state-of-the-art developments in biotechnology. Considering the aforementioned trends in new computational methods and advances in classical informatics for translational applications of these methods, these reviews can be complemented by a dedicated discussion restricted to *in silico* approaches for NP drug discovery.

Another trend in drug discovery enabled by informatics and computational methods is an increasing shift toward a data driven drug discovery (Tatonetti et al., 2012; Lusher et al., 2014). Traditionally, drug discovery has been performed as follows: basic scientists first find a target structure in the human body related to a disease or illness, followed by screening for “lead” compounds that show affinity for the target. Subsequently, the list of candidates is narrowed down (using some of the methods described in this review) to find the most promising leads, which then go through the development process to assess safety and efficacy in model organisms and, eventually, humans. A detailed description of these steps can be found in other reviews (Hughes et al., 2011). Failure at any stage in this workflow can—and usually does—necessitate starting over from the beginning, contributing to the estimated cost of 2.6 billion USD to bring a new drug to market (Avorn, 2015). Data-driven drug discovery turns the process on its head, by using data mining on large data repositories of candidate compounds and disease knowledge to generate novel therapeutic hypotheses systematically rather than hoping for a single therapeutic hypothesis to deliver actionable results. Aside from avoiding systematic biases present in the hypothesis-driven model, this additionally helps to improve the return rate on subsequent manual experimentation and validation of lead compounds, ultimately lowering costs and increasing productivity (Jorgensen, 2004). Data-driven drug discovery leverages new data types that were previously inaccessible, and relies heavily upon computers and informatics techniques to produce increasingly accurate results (Butte and Ito, 2012).

**Figure 1:**
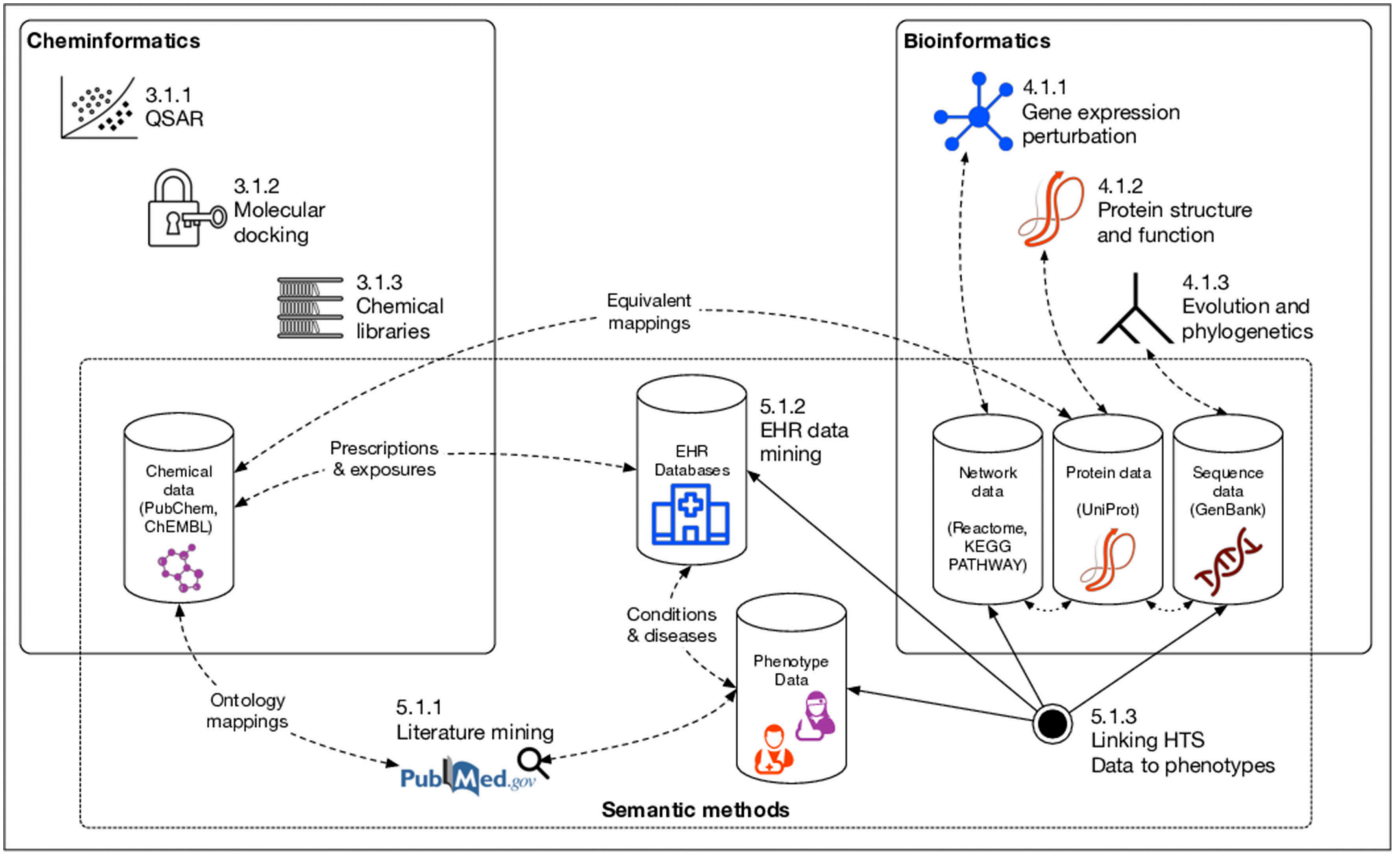
Informatics methods for natural product drug discovery covered in this review. Numbers preceding methods correspond to section/subsection numbers in the manuscript describing the method. Dashed lines indicate inferred links between various data resources.

In this review, we first discuss various major classes of natural products based both on source organism and their biological functions. In addition, we provide examples of specific members of those classes with demonstrated therapeutic potential. We then explore several major disciplines based upon informatics and computational methods—cheminformatics, bioinformatics, and semantic (or “knowledge-based”) informatics—and their associated methods that can be used specifically for NP drug discovery. These methods are summarized graphically in Figure 1. Finally, we conclude with a recap of the major gaps currently facing the field of computational NP drug discovery, and suggest actions for the future that could help to resolve these problems.

## 2. Classes of Therapeutic Natural Products

There is no definitive consensus on what groups of substances comprise “natural products,” with some authors restricting them to small molecule secondary metabolites (Nature Publishing Group, 2007), and others more broadly stating that an NP is any chemical substance produced by a living organism (National Center for Complementary and Integrative Health, 2017). For the purpose of this review, we adopt the latter of these two definitions: that natural products include all classes of chemical substances that are produced or recruited by living organisms, and have the ability to be isolated and reused by humans. This definition includes an incredibly diverse range of compound types; therefore, it is crucial to understand the different subgroups of NPs, along with their characteristics. These classes of NPs frequently overlap and have vaguely defined boundaries, but they are nevertheless useful for understanding the methods that can be applied to them.

### 2.1. Phytochemicals

Phytochemicals—chemicals synthesized by plants—encompass a broad range of NPs, including members of many of the other classes described later in this section. Phytochemicals can be toxic, they can provide important dietary nutrients (such as amino acids, antioxidants, and dietary fiber), or they can be inert in humans. For most research purposes, however, phytochemicals are limited to primary and secondary metabolites in plants, which can be generally divided into phenolic acids, stilbenes, and flavonoids (which, themselves, can be further subdivided into more specific subclasses), all of which are small molecules (rather than macromolecules, which tend to be prevalent in many of the other classes we discuss) (Harborne, 1999). These chemicals have been the source of many traditional and modern medicines, famous examples of which include the analgesic acetylsalicylic acid (aspirin), the heart medication digoxin, and the chemotherapy drug paclitaxel (Molyneux et al., 2007).

### 2.2. Fungal Metabolites

Fungal metabolites serve a relatively similar role to plant metabolites, so much so that they share some of the same subclasses (perhaps most notably the flavonoid compounds). Like plant metabolites, fungal metabolites can treat a wide variety of diseases and conditions, but they are perhaps most famous as a source of many successful antibiotics. Other areas of successful application include antimalarials (antiamoebin), immunosuppressants (ciclosporin), statins (mevastatin, lovastatin), and more (Thomford et al., 2018).

### 2.3. Toxins

Toxins are substances that can potentially harm or kill. They include poisons and venoms, and are (by definition) produced by living organisms. Poisons are toxins that cause harmful effects when swallowed, inhaled, or absorbed through the surface of the skin, while venoms are toxins that cause harm when actively injected via a sting or a bite.

Poisons are produced by members of many major clades of organisms, including plants, fungi, bacteria, and most groups of animals. Natural poisons are usually used for defensive purposes, although some species have adapted them for more complex roles (Klaassen and Watkins, 1996). They can include members of all classes of molecules, and although many tend to consist of relatively small molecular structures, macromolecules, such as proteins, large carbohydrates, and lipids can be poisonous as well. NP poisons include many chemotherapy drugs, particularly when their toxic effects act more selectively on cancer cells than healthy cells. Some examples include paclitaxel (from *Taxus brevifolia*) and vinblastine (from *Catharanthus roseus*) (Thomford et al., 2018).

Venoms are complex mixtures of chemicals produced by animals for either defensive or offensive purposes (or, sometimes, both in the same species). An individual species' venom can include hundreds of unique chemical compounds, many of which are proteins that act on specific molecular targets. Venoms are highly evolutionarily optimized to fit organisms' biological niches (Daltry et al., 1996), but due to interspecies homology, the effects of individual venom components have led to numerous therapeutic applications, including FDA-approved treatments for hypertension, diabetes, neuropathic pain, and more (Lewis and Garcia, 2003). Like poisons, venoms have also demonstrated potent anti-cancer effects, and their high target specificity has made them of particular interest for applications of precision medicine, particularly for rare or aggressive cancer types (Romano and Tatonetti, 2016; Yang et al., 2018).

### 2.4. Antibodies

Components of the immune system—particularly antibodies—have long been attractive for drug discovery and design. Their primary function is recognition and inactivation of pathogens, including bacteria and viruses, but biotechnologists have repurposed them for many “unintended” uses, including the targeted treatment of various diseases. One approach, known as immunotherapy, involves the design and application of monoclonal antibodies that bind specifically to certain cells or proteins related to the disease of interest. Naturally, these are often autoimmune diseases, such as rheumatoid arthritis (Seo et al., 2004) and allergies (Jutel et al., 1995), but they have also been applied to diverse diseases, such as viral infections (Letvin and Walker, 2003) and multiple sclerosis (Hohlfeld, 1997). Recently, substantial attention has been given to immunotherapy treatments for cancer, exemplified by the 2018 Nobel Prize in Medicine being awarded for research in this area (Ishida et al., 1992; Leach et al., 1996; Rosenberg et al., 2004). The second approach involves using antibodies as delivery agents for therapeutic compounds, which is also being explored extensively for cancer, due to its capacity to mitigate off-target effects (Awwad and Angkawinitwong, 2018). Interestingly, this delivery method has attracted specific attention for the delivery of chemotherapeutics that are, themselves, NPs (Mann, 2002).

It should be noted that—in spite of the substantial accomplishments described above—antibodies have failed to deliver on several therapeutic applications that originally held promise, often for characteristics that are inherent to antibodies in general. One example involves the treatment of Alzheimer Disease (AD) using monoclonal antibodies. Antibody-based treatments for AD performed strongly in mouse models (Bard et al., 2000) and in early-phase clinical trials (Hock et al., 2003), but in phase-2 trials and beyond, they have failed to deliver (Tayeb et al., 2013). Multiple theories have been posed, but the two leading hypotheses for failure have been that (1) antibodies are limited in their ability to cross the blood-brain barrier, and (2) certain degenerative diseases require early treatment for antibodies to be effective, far before patients begin to show symptoms (Sperling et al., 2011). Other failures in antibody therapy are related to the activity of antibodies themselves—drugs like theralizumab (designed to treat leukemia and rheumatoid arthritis) failed in human trials due to inciting a life-threatening “cytokine storm” in all healthy volunteers (Eastwood et al., 2010). Nonetheless, much research on new antibody therapies is being conducted to treat the same diseases associated with these early failures (Sevigny et al., 2016).

### 2.5. NPs With Limited Therapeutic Potential

The classes of NPs described above cover substantial breadth. However, to provide a more complete image of drug discovery in terms of NPs, it is also important to consider classes with only limited—or at least presently unknown—therapeutic potential. For the purposes of this review, we focus on whether a compound is reactive enough in living systems to potentially perturb that system. If it is, then there exists an opportunity to exploit the perturbations for potentially therapeutic outcomes. The largest group of NPs that falls short in this regard is those with purely structural purposes, including materials like wood, biopolymers, and excretions like spider silk, which suggests that the drug discovery methods discussed in subsequent sections of this review are unlikely to generate many new lead compounds.

Nonetheless, biology is rife with exceptions to every rule, and even these groups of NPs have occasionally yielded compounds with therapeutic use. Wood creosote has been used for centuries as a treatment for diarrhea, and is currently marketed in Japan under the trade name Seirogan (Hiramoto et al., 2012). Biopolymers have not resulted in drugs themselves, but have been used many times to successfully deliver drugs within living systems (Nitta and Numata, 2013). Even spider silk has shown potential in drug delivery (Spiess et al., 2010), and has been bioengineered to have antibiotic properties (Harvey et al., 2017). For this reason, we hesitate to say that any class of NPs has no therapeutic potential. In a practical sense, these observations are most useful in a cost-benefit analysis scenario, when it is necessary to balance research budget with scientific risk, highlighted by Dickson and Gagnon as one of the major factors influencing the total output of the pharmaceutical industry (Dickson and Gagnon, 2004).

## 3. Cheminformatics Methods

Cheminformatics methods can generally be classified according to the types of characteristics they exploit: either direct measures of chemical activity (e.g., chemical constants, reactive groups, or ADME measurements), or indirect measures (e.g., structural motifs, compound class membership, or other higher-order observations). These techniques can be further subdivided; for example, structural comparisons can be applied either before or after promising chemical activity is known (which we refer to here as prospective and retrospective structure mining, respectively). Prospective structure mining is conducted in a supervised manner, where known chemical activity of well-characterized compounds is compared to the structures of query compounds to predict the therapeutic potential of the queries. Retrospective structure mining, on the other hand, is more analogous to unsupervised learning techniques, where other screening techniques first identify a compound of interest (referred to as a “hit”), and then seek to expand the number of candidate compounds by searching for structures that are similar to the hit compound.

### 3.1. Cheminformatics and Natural Products

Many traditional cheminformatics methods are challenging to adapt to certain classes of NPs, particularly when the NPs consist of large chemical structures (like venoms, antibodies, or other protein-based NP drug candidates). For example, generating combinatorial libraries of large polypeptides is currently intractable, due to the massive search space. However, additional characteristics that are unique to these classes of NPs enable either simplifying assumptions to be made or the invention of entirely new approaches for predicting bioactivity (Huang et al., 2016). Here, we divide cheminformatics into 3 major categories of methods that have been used to success with NPs, providing discussion of the caveats that must be considered for NPs in particular.

#### 3.1.1. Natural Product QSAR Analysis

Quantitative Structure Activity Relationship (QSAR) analysis is a widely used—if often ambiguously defined—technique in cheminformatics for predicting a response variable given a set of structural, chemical, and or physical input variables (known as *molecular descriptors*). Generally, the goal is to learn a function of the form

ŷ=f(x)+ϵ

where **x** = (*x*_1_, …, *x*_*N*_) is the vector of *N* input variables, ŷ is the estimated response (continuous in the case of regression, and integer-valued in the case of classification), and ϵ is an error term. *f* can be any appropriate model; common choices include logistic regression, support vector machines, random forest, artificial neural networks, and others. Recently, deep learning has shown to be particularly effective for predicting a wide variety of responses, including solubility, probe-likeness, and others (Korotcov et al., 2017). A number of free and commercial software implementations of QSAR are available for a variety of use cases (Benfenati et al., 2011; Tosco and Balle, 2011), and approaches for adapting generic statistical and machine learning models for QSAR are readily available (Lavecchia, 2015).

QSAR has been applied fairly widely to different classes of NPs, where specific classes tend to dictate the chosen molecular descriptors. Typical choices for non-NP applications include symbolic (1- or 2-D) descriptors, 3-D spatial organization, higher-order (e.g., time-dependent or ligand-bound) conformational characteristics (Polanski, 2009), experimental measurements (partition coefficient, polarizability, refractivity, etc.), and many others. For a detailed review of these and similar descriptors, see Cherkasov et al. (2014). Additional characteristics that can be used for small-molecule NPs include categorical (“one-hot”) variables indicating class membership (e.g., alkaloid, terpenoid), species of origin (or more general taxonomic clades), and other biological features. Macromolecular NPs are substantially more restricted in terms of the types of descriptors that can be used effectively. Generally, 3-D conformational descriptors and binding data function best for these NPs, and yield good results (Mladenović et al., 2017; Dhiman et al., 2018). QSAR has performed adequately for predicting binding affinity of antibodies to proteins—Mandrika et al. describe a model consisting of 26 physicochemical descriptors (covering hydrophobicity, polarity, electronegativity, etc.) at each amino acid position in a library consisting of single chain monoclonal antibodies (Mandrika et al., 2007). While this model has not yet been applied to NP drug discovery, it seems to be a feasible way forward.

#### 3.1.2. Molecular Docking and Dynamics

QSAR is a useful statistical method for predicting potentially therapeutic interactions, but it is often desirable to directly model the chemical or physical interaction that is being investigated. Molecular docking is an approach that seeks to predict if and how two compounds (usually a target and a ligand) physically interact. This is usually performed in two steps: (1) searching for potential conformational fits, and (2) scoring those fits. Molecular dynamics is a particular simulation technique that can be applied to docking, and is popular in drug development. From a high level, molecular dynamics performs a computational simulation of the atoms and molecules (often including solvents) present in a putative reaction, and allows the molecules to interact for a period of time. The technical details and algorithms for docking and dynamics are well-summarized elsewhere (Karplus and McCammon, 2002; Pagadala et al., 2017)—we will instead focus on broad caveats, issues, and innovations in applying these to NPs.

The class of NP compound tends to dictate the role (target vs. ligand) that the compound plays in docking simulations. Typically, small molecule NPs and relatively short polypeptides (e.g., peptide toxins and venom components) act as ligands, while larger proteins and protein complexes act as targets (although exceptions are common). This distinction is important, especially when the goal is screening many candidate compounds: usually, the target is held fixed, while the ligand can be drawn from libraries of many compounds. Therefore, it is computationally feasible to perform docking of many small molecule compounds when a specific molecular target is already known (Khan et al., 2009; Lee et al., 2011; Ma et al., 2011). Conversely, if a macromolecular NP is suspected of interacting with endogenous small-molecule metabolites (e.g., in human cancer cells), docking simulations can be used to mine *which* metabolites could bind to the NP (Pithayanukul et al., 2009). If both a target and a ligand are already predicted by other means (e.g., QSAR or other methods described in this review), docking is commonly used as a secondary validation method. In spite of their large molecular weight, antibodies are relatively easy to screen in large numbers via docking, due to their specific structural and binding constraints that can substantially reduce computational complexity of simulations (Walls and Sternberg, 1992; Abagyan and Totrov, 2001).

Molecular dynamics is an important technique for characterizing physical interactions of putative drugs with their targets, but due to computational challenges it cannot be used with current technologies in a data-driven manner to screen very large numbers of NPs against similarly large numbers of potential targets simultaneously (Salmaso and Moro, 2018). However, it has proven incredibly valuable in uncovering specific therapeutic mechanisms of NPs (venom proteins in particular). An early and influential example of this came in 1995, when Albrand et al. combined molecular dynamics with NMR to explain how Toxin FS2 (from Black Mamba venom) blocks L-type calcium channels, causing potent cardiotoxic effects (Albrand et al., 1995). Additionally, there are noteworthy success stories that have emerged from screening relatively small NP databases against specific drug targets: The compound ellagic acid—which has shown both antiproliferative and antioxidant properties—was identified by Moro et al. by screening a proprietary database of 2,000 NPs against the oncoprotein casein kinase 2 (Cozza et al., 2006). Similarly, Fu et al. identified Jadomycin B—another molecule with anticancer effects—by screening 15,000 microbial small molecule metabolites against the oncoprotein Aurora-B kinase (Fu et al., 2008). These examples illustrate the feasibility of molecular dynamic studies for discovering new therapeutic NPs, and suggest that overcoming associated computational challenges will enable their widespread application in diverse and data-driven contexts.

#### 3.1.3. Computational Mutagenesis and Library Construction

One of the most common techniques for identifying drug candidates is to generate massive libraries of compounds that can be screened in parallel, with the understanding that only a very small fraction will result in “hits” (potential therapeutic activity). There are many ways such libraries are generated, many of which fall under the umbrella term of combinatorial chemistry (i.e., enumerating chemical structures using combinatorics) (Terrett et al., 1995). NPs provide some advantages over traditional (non-NP) classes of candidate compounds, namely that such “libraries” already exist in nature. General purpose online databases of chemical compounds (such as PubChem and ChEMBL) (Li et al., 2010; Gaulton et al., 2016) contain many NPs that are annotated by compound class, while other, more specific databases (such as ArachnoServer, VenomKB, and the Dictionary of Marine Natural Products) provide even more granular annotations for aggregating NP libraries with various characteristics of interest (Pineda et al., 2017; Romano et al., 2018).

Computational mutagenesis is a related class of techniques that has shown efficacy in certain classes of NPs. This method involves specifying a template (e.g., a certain antibody with putative therapeutic activity that requires optimization), and then sequentially mutating locations in the template's structure to generate a library of candidate compounds. These libraries can then be screened *in silico* (e.g., using molecular docking simulations as described in section 3.1.2) to find structures that can be engineered in the lab. Antibodies, in particular, are particularly well-suited to computational mutagenesis, by modifying amino acids in binding regions (Sivasubramanian et al., 2009; Wollacott et al., 2019). The feasibility of mutagenesis techniques in the context of NP drug discovery was demonstrated by Chen et al., who generated a library of analogs of the 7-residue NP peptide HUN-7293 to optimize its inhibitory effects on cell-adhesion (Chen et al., 2002).

It should be noted that one of the advantages of working with NPs is the potential of avoiding library screening entirely, under the assumption that nature has optimized it for biological activity. This point is expanded on in section 4.1.3.

## 4. Bioinformatics Methods

Bioinformatics methods for drug discovery include anything related to the *biological* function of potential drug candidates, including sequence-based characteristics, interactions with body structures (metabolites, proteins, cells, tissues, etc.), pathway perturbations, and toxicity, among others. Multi-omics and high-throughput sequencing are also major areas within bioinformatics. Most subdisciplines of bioinformatics can be applied in some way to the drug discovery process (Wishart et al., 2017; Thomford et al., 2018).

### 4.1. Bioinformatics and Natural Products

In the case of NPs, researchers are able to make use of an entire range of techniques related to the organisms that produce the compounds. In particular, phylogenetics and evolution provide many routes for various drug discovery activities. Closely related organisms often produce similar proteins and metabolites, so when one natural compound with promising activity has an unsuitable therapeutic index for human use, libraries of similar compounds can be easily constructed by searching in organisms within the same genus. However, these techniques must be applied with caution: members of some groups of natural compounds (such as venom proteins) are heavily optimized to fit a very particular biological niche, so even members of the same species may have entirely unique metabolic profiles with respect to compounds of interest. One prominent example of this was found in the rattlesnake species *Crotalus oreganus helleri*, where members of the species living on different sides of a mountain range produced entirely separate venom profiles (Sunagar et al., 2014).

#### 4.1.1. Gene Expression Perturbation

The rise of multi-omics approaches to uncovering mechanisms of disease has led to multitudes of ways to assess the effect that putative drugs have on cells. In particular, gene expression perturbation—quantified using RNA-sequencing and transcriptomics—has led to a number of innovative breakthroughs in drug discovery for diseases associated with gene disregulation, including cancers and various other diseases with complex genetic etiologies (Sirota et al., 2011; Subramanian et al., 2017). Along with environmental exposures, structural abnormalities, and other influencing factors, these diseases often can be attributed in part to abnormalities in gene expression, including the systems-level effects of expression perturbation in the larger context of cell signaling and metabolic networks (Nica and Dermitzakis, 2008; Cookson et al., 2009). More accurately, differential expression can be treated as a phenotypic signal that arises from underlying disease etiology. Accordingly, drugs and drug candidates that effectively invert such deleterious effects are potential therapies for these diseases.

This technique is particularly well-adapted for use in NP drug discovery, as vast numbers of compounds from all classes of NPs are specifically optimized to have roles in cell signaling or metabolic networks, and are already known to be relatively biologically stable (Lewis and Garcia, 2003). Compounds used in Traditional Chinese Medicine (TCM) have been particularly well-utilized in this area. In a 2014 study, researchers uncovered likely mechanisms by which the TCM compound berberine exhibits anti-cancer activity, using publicly-available expression data for berberine-perturbed human cells taken from the Connectivity Map (CMap) project (Lee et al., 2014). Another important recent example by Lv et al. provides differential gene expression profiles in response to 102 different TCM compounds, presented as a framework from which to base future systematic research on the activities of TCMs (Lv et al., 2017).

A separate but related approach involves analysis of differential expression in the organisms producing the NPs (rather than the organisms that NPs act upon). An investigation by Amos et al. discovered previously unknown NPs—as well as putative mechanisms describing their functionality—by comparing transcriptome profiles of different bacterial species in the genus *Salinispora* (Amos et al., 2017), underscoring the diversity of emerging multi-omics techniques that can be employed within NP drug discovery.

#### 4.1.2. Modeling Protein Structure and Function

Although the size and complexity of proteins is often prohibitive to structure-based analyses designed for small molecules, other drug discovery approaches leverage the unique characteristics of proteins and other macromolecules to perform discovery in ways that are otherwise impossible. Since many classes of NPs are comprised of proteins, these techniques can often be adapted to NP drug discovery with relative ease.

Some methods use supervised machine learning algorithms trained on protein structures (and motifs) with known activity to predict activity in new, uncharacterized proteins—this is essentially traditional QSAR designed to work on proteins. The FEATURE framework (Halperin et al., 2008) does this using 3-dimensional spatial orientation of atoms to predict activity at numerous “microenvironments” within a larger macromolecule, and is therefore generalizable to diverse proteins with conserved functional activity. Other research teams have designed similar frameworks based on other machine learning models, including deep learning models like convolutional neural networks (Torng and Altman, 2017; Thomas et al., 2018). For further details on learning protein function from structure, we refer the reader to Pérez et al. (2018).

Still other protein functional modeling approaches rely on input variables that behave like “abstractions” of raw molecular characteristics, including amino acid or DNA structure (along with sequence alignment algorithms) (Vyas et al., 2012), ontology annotations (see section 5 for more details) (Mutowo et al., 2016), and biomarker response (Frank and Hargreaves, 2003).

#### 4.1.3. Using Evolution to Discover Drug Candidates

The fact that NPs are derived from living organisms implies that they either serve a specific purpose in the context of that organism, or they are a byproduct of an important process (Stone and Williams, 1992). Therefore, we can use evolution and taxonomy as tools for both discovering new compounds and their effects, as well as for generating libraries of similar natural products (Maplestone et al., 1992).

The simplest—and most common—use of phylogenetics in natural product drug discovery revolves around the axiom that *closely related species produce similar NPs*. This can be used to predict the structures of NPs, given structures for similar NPs in related species are already known (Ziemert and Jensen, 2012). Following a pattern akin to QSAR modeling (described in section 3.1.1), phylogenetics can also be repurposed to predict other characteristics of closely related NPs, including molecule classes, toxicity, stability, and others, where instead of using molecular descriptors as observed features of the NP, you instead use evolutionary characteristics to build a predictive model. A noteworthy example is given by Malhotra et al., who used discriminant function analysis (DFA) to classify and predict functions of over 250 phospholipase A_2_ proteins from viperid snakes, where aligned amino acid sequences alone were used to construct the input features for the DFA model (Malhotra et al., 2013).

Other uses of evolution in drug discovery employ phylogenomics to discover associations across more distantly related species (e.g., between humans and microbes). This includes efforts to catalog the entire breadth of various classes of natural products to create comprehensive NP class libraries (see section 3.1.3 for more details) (Rønsted et al., 2012). In 2016, Rudolf et al. showed that comparative genomics in diverse microbial species could identify 87 distinct gene clusters across 78 bacterial species corresponding to a class of putative NP anticancer drugs known as enediynes (Rudolf et al., 2016). By finding instances of NP coevolution in distantly related species, studies have uncovered compounds that play keystone roles in metabolic processes, leading to therapeutic solutions in analogous processes in humans. A noteworthy and sophisticated example is shown in the CSMNA method (Zhang et al., 2016), which is based on the hypothesis that similarities between human and plant metabolic networks can be used to guide phytochemical drug discovery. The authors validate their drug discovery algorithm by showing that similarities between the plant Halliwell-Asada (HA) cycle and the human Nrf2-ARE pathway underlie antioxidant activity of HA cycle molecules on proteins in the Nrf2-ARE pathway.

Some caveats need to be kept in mind when using evolutionary approaches. Certain classes of NPs are under evolutionary pressures that complicate phylogenetic analysis. Venom proteins, in particular, can be highly divergent even among species within the same genus (Calvete et al., 2014), a phenomenon attributed to the high metabolic cost of venom production, and the highly targeted nature of many venom proteins to specific prey species.

## 5. Semantic (Knowledge-Based) Methods

Cheminformatics and bioinformatics are both major subdivisions of biomedical informatics, and comprise two of the primary disciplines involved in translational research and drug discovery. We now turn our focus to a set of methods that emerged from semiotics, linguistics, and library science, but have been adapted to serve broad functions in computer science and artificial intelligence—known as knowledge-based or semantic (i.e., relating to human-interpretable meaning) methods. In general, these are methods involving the application of various knowledge representations, such as ontologies and structured terminologies. Some activities within this group include rule-based natural language processing, certain types of clinical data mining, knowledge extraction, semantic data normalization, and others. Especially in the context of drug discovery, knowledge-based methods are frequently applied in coordination with bioinformatics and/or cheminformatics methods, and serve as one of the main approaches to combining and unifying findings and intermediate results spread across separate research activities.

Perhaps the most well-utilized resource in knowledge-based approaches to drug discovery is the Gene Ontology (Ashburner et al., 2000), which classifies conceptual biological entities into 3 groups: molecular functions, cellular components, and biological processes (each of which is important in various stages of the drug discovery process). Researchers have created multitudes of data resources to assist in drug discovery, and many of these are mapped to the Gene Ontology to assist with *in silico* aggregation and preliminary validation of putative hypotheses. Some of these linked resources include DrugBank (Wishart et al., 2017), UniProtKB/Swiss-Prot (and associated annotation programs like ToxProt) (Jungo et al., 2012), and ChEMBL (Gaulton et al., 2016), all of which catalog compounds that may confer some therapeutic effect.

Still other tools have been created to map unstructured data relevant to drug discovery (such as journal article abstracts in PubMed) to more structured representations. MetaMap, SemRep, and Semantic Medline from the National Library of Medicine, as well as the NCBO Annotator from the National Center for Biomedical Ontology identify ontology and terminology terms within free text (usually pulled from journal articles) at various levels of abstraction. These tools have been used to successfully perform ontological inference across multiple levels of evidence for many discovery tasks, including drug discovery. For further details, we refer the reader to the original paper describing Swanson's Fish Oil-Raynaud's Syndrome hypothesis (Swanson, 1986), which explains how structured knowledge and graph algorithms can be used to discover informative associations fragmented across otherwise unrelated publications (Cameron et al., 2013).

Other levels of knowledge representation (e.g., not formally controlled at the concept level) also have important roles in drug discovery; tools like OMIM can be used to map newly discovered drug-gene associations to diseases that are modulated by that gene or set of genes. For comprehensive listings of the various ontologies, knowledge representations, and similar tools with proven roles in drug discovery, we refer the reader to a number of existing reviews (Gardner, 2005; Vazquez-Naya et al., 2010; Thomford et al., 2018).

### 5.1. Semantic Methods and Natural Products

While the number of ontologies and similar resources relevant to drug discovery are vast, advanced applications of these resources are relatively scarce. This trend is even more striking in regards to NP drug discovery. As of now, most therapeutic associations between NPs and disease are discovered serendipitously rather than through systematic, rigorous applications, although earlier sections of this review describe notable exceptions to this trend. In light of the fact that advanced use of semantic methods is rare in NP drug discovery, we will additionally consider applications of ontologies and terminologies used for drug discovery that *could* be applied to NPs, based on current knowledge.

#### 5.1.1. Literature Mining

Literature mining—the process of performing text mining on scientific literature databases—is one of the most common usages of semantic biomedical knowledge resources. The MEDLINE/PubMed database contains over 26 million biomedical text citations, many thousands of which contain knowledge related to NPs, and possibly describing characteristics of those NPs that provide direct or indirect evidence of therapeutic activity. There are generally two ways to automatically extract such knowledge from biomedical publications: (1) Using existing ontology/terminology annotations, or (2) using natural language processing (NLP) techniques that discover such annotations.

Medical Subject Headings (MeSH) are one terminology resource designed to structure the content of PubMed articles, and are applied manually by expert annotators at the US National Library of Medicine (NLM) to new articles shortly after indexing in PubMed (Lipscomb, 2000). MeSH terms cover a diverse range of biomedical concepts, arranged in a hierarchical fashion, and cover various classes of NPs. MeSH can be used to aggregate PubMed articles describing certain types of NPs, and can be refined using additional terms (e.g., “Drug Discovery”) or qualifiers (e.g., “/therapeutic use”). MeSH terms can link journal entities to structured external databases by either using cross-mappings [including via the NLM's Unified Medical Language System (UMLS)] or annotations in external databases directly to MeSH terms (Ruau et al., 2011). MeSH terms have been used to summarize components of plant genomes (Beissinger and Morota, 2017), demonstrating potential paths forward in discovering novel NPs (rather than using the terms to gather knowledge about known NPs).

A limited number of databases provide access to curated sets of articles describing NPs. VenomKB provides articles annotated to venom components as well as literature predictions describing the putative therapeutic effects of those components and mappings to other external databases (Romano and Tatonetti, 2015). Similarly, NPASS presents chemical characteristics of a broader range of NPs and provides references to PubMed entries describing manually-curated biological activity measurements in a range of organisms (including humans) (Zeng et al., 2017). Other databases, including MarinLit and NAPRALERT, provide commercial and paid access to curated NP literature data.

#### 5.1.2. Electronic Health Record Mining

Similarly to literature mining, we can apply knowledge retrieval techniques to observational data sources. As far as drug discovery is concerned, observational data provides a method for assessing the effects compounds have on humans in the absence of rigorously controlled clinical research studies. This style of data analysis offers several major advantages over clinical trials, including avoidance of exposing new patients to potentially harmful treatments, and mitigating certain types of bias associated with eligibility and patient selection. Observational data can often produce larger cohorts than clinical trials. Various sources of observational data can be utilized for drug discovery, but here we will focus on electronic health records (EHRs), due to their prevalence and proven utility for many translational research tasks. Although privacy concerns, data fragmentation, and standardization have traditionally hampered access to EHR data—particularly for research teams without clinical expertise or affiliation with a large academic medical center—rapidly growing efforts, such as Observational Health Data Sciences and Informatics (OHDSI) (Hripcsak et al., 2015) and the Electronic Medical Records and Genomics (eMERGE) network (McCarty et al., 2011) are breaking these barriers in ways that will increase access to data covering the breadth of the translational spectrum.

EHR data are complex, multimodal, and subject to many unique biases and ethical/legal constraints (Weiskopf and Weng, 2013). In addition to free text (recorded by health care providers), a number of structured data types are also present (including claims data, medication orders, laboratory measurements, patient demographics, and others). As of now, no major applications of EHR data mining to NP drug discovery have been reported, but a number of related areas provide hints as to its feasibility. A review by Yao et al. highlights 3 specific ways that EHRs can aid drug discovery: (1) Finding relationships between diseases for the purposes of drug repurposing, (2) evaluating the usage patterns and safety of drugs and/or drug candidates, and (3) discovering phenotype–genotype associations that can lead to the discovery of new drug targets for specific diseases (Yao et al., 2011). Relevant caveats of each of these can be discussed from the perspective of NP drug discovery, including specific advantages and disadvantages that NPs provide when compared to non-NP drugs and drug candidates.

Drug repositioning involves taking an existing drug and using it to treat a different disease than what it is currently intended for Ashburn and Thor (2004). EHRs have been used for a number of drug repositioning approaches. The most common repositioning strategy involves discovering similarities between diseases, and then using those similarities to imply new treatments. This is based on the assumption that diseases with similar etiologies will produce similar signals in the EHR, and that similar etiologies may imply similar treatments. An important example by Rzhetsky et al. showed unexpected similarity between bipolar disorder and breast cancer (Rzhetsky et al., 2007). Recently, it has been demonstrated that the breast cancer drug tamoxifen may be useful for treating the symptoms of bipolar disorder (Kulkarni et al., 2006).

EHR data can also be used to assess the safety of drugs (or putative drugs), by determining whether exposure to the drug increases risk of adverse effects (Schuemie et al., 2012; Tatonetti et al., 2012). This is easiest for approved drugs that have coded representations in the EHR software (e.g., those with ATC codes or similar—experimental and unapproved drugs generally do not have a structured representation in EHR databases), but natural language processing can identify experimental and putative drugs with reasonable efficacy (Björne et al., 2013). This suggests that NP drug-candidate safety surveillance could be performed on free-text notes in the EHR, especially when treated as environmental exposures rather than physician-prescribed interventions. The feasibility of this approach was demonstrated by Zhang et al., who showed that herbal and natural supplements (which are usually considered NPs) could be identified in medication lists using natural language processing, and quantified the gap between structured drug representations and these compounds (Zhang et al., 2015). Two of the main gaps in need of resolution to realize this goal include specifying a standardized nomenclature for NPs (Dewick, 2002), and identifying where (geographically) hospital patients may be exposed to the NPs being investigated.

Discovering new drug targets is not strictly the same thing as drug discovery, but it does provide an essential starting point for identifying new drug leads. Recent decades have seen a steady decline in the discovery of new targets, and previous reviews on the topic have called for new and innovative strategies to address this issue (Lindsay, 2005; Spedding, 2006). Using EHR data and clinical biobanks to conduct Genome Wide Association Studies (GWASs) and Phenome Wide Association Studies (PheWASs) are touted as solutions (Yao et al., 2011), by providing associative links between diseases and specific genetic loci, which can then be used as targets for new precision drug therapies (McCarty and Wilke, 2010; Wilke et al., 2011). NPs, in particular, come into play when considering their unique abilities to target certain genes and gene products that are poorly targeted by small molecules. Both monoclonal antibodies and protein-based therapeutics are known for their ability to target individual cell types, especially useful in cancers with specific genetic signatures (Adams and Weiner, 2005; Cox et al., 2016). GWAS and PheWAS are relatively new compared to the drug discovery and development timeline, but we will likely see many NP drugs emerging from clinical trials that used EHR- and biobank-enabled analyses for target discovery in the coming decades (Thomford et al., 2018).

#### 5.1.3. Linking HTS Data to Putative Disease Treatments

Until now, we have discussed ways that ontologies and terminologies can be used to retrieve and structure knowledge, but another important role semantic techniques play in biomedicine is integrating disparate data sources in ways that otherwise require massive amounts of manual interpretation and annotation to apply at scale. This is important for many reasons, including experimental validation, increasing statistical power and inferential capacity, and even to discover new knowledge entirely. A particular application that has experienced rapid growth and major methodological advancements in drug discovery is linking new types of high-throughput sequencing (HTS) data to clinically-meaningful associations. Previously mentioned techniques, such as gene expression perturbation (section 4.1.1) yield results consisting of signals that have biological meaning, but no explicit connection to clinical phenotypes. Important early examples of data-driven drug discovery from gene expression formed therapeutic associations between cimetidine and lung adenocarcinoma (Sirota et al., 2011), as well as topiramate and inflammatory bowel disease (Dudley et al., 2011), but these examples required manual curation of many phenotype-linked expression profiles from which discovery could be performed. Knowledge representations provide a method for making these connections automatically, when correctly leveraged.

Successful knowledge integration of this type requires links to be formed between (a.) sets of genes (or, more specifically, groups of probe sets) and metabolic pathways, as well as (b.) links between pathways and phenotypes. A number of well-established and richly annotated gene-pathway databases (including Reactome and KEGG) (Fabregat et al., 2015; Kanehisa et al., 2016) already exist, and are used widely by the biomedical research community. Resources linking pathways to phenotypes are considerably less prevalent (and less complete), due largely to a limitation of available, relevant data, but ongoing efforts in the translational bioinformatics community are changing this. Integrating differences in gene expression and phenotypic response at the cell- and tissue-level with pathway data has shown particular promise in this area (Hao and Tatonetti, 2016; Hao et al., 2018). A recent review by Oellrich et al. outlines emerging and established tools for computational phenotyping (Oellrich et al., 2015).

Similar studies are, however, nearly absent from the realm of NP drug discovery. The unique characteristics of different NP classes (especially those described earlier in this review) can facilitate the phenotyping process. Metabolomics data provides clues as to NPs' original functions in their source organisms, which can often be extended to their effects when applied to humans (Xie et al., 2008; Yan et al., 2015; Zhang et al., 2016). Phylogenomics can highlight similarities between the genetic epidemiologies of complex diseases in humans vs. model organisms, possibly suggesting species from which to mine compounds that can treat these diseases (Romano et al., 2015). Even the predator/prey adaptations of NP-producing species can suggest the biological function of NPs (de la Vega and Possani, 2005; Miller et al., 2016); the discovery that the cone snail *Conus geographus* hunts fish by releasing insulin into the surrounding water (resulting in rapid hypoglycemic shock in the prey) led to the identification of a powerful insulin-receptor-binding motif that has shown considerable promise for future treatments of diabetes (Menting et al., 2016). Some recent studies focusing on discovery from TCM data show promise: Cui et al., for example, created a TCM chemical structure database that they screened against acetylcholinesterase (ACE) inhibitors, both via docking simulations with the known structure of ACE, as well as similarity to existing ACE inhibitors retrieved from BindingDB (Cui et al., 2015). Conceivably, ontology resources could be used to adapt these methods into an automated approach for screening many drug classes with little to no manual curation.

Linking HTS data to disease phenotypes is only one application of semantic knowledge resources that could be a boon for NP drug discovery. There are many other conceivable uses for linking evidence between clinical datasets, drug terminologies, literature-mined associations, and organismal biodiversity data, any of which could lead to potentially valuable discoveries and improved evidence for unproven hypotheses.

## 6. Gaps and Opportunities

### 6.1. Comparing the Use of Informatics Disciplines in NP Drug Discovery

Computers have revolutionized the way medicine and biomedical research are conducted, and the same applies to drug discovery. In doing so, it is critical to consider all of the ways in which computers can assist the discovery process in order to maximize the return on research efforts. In terms of *natural product* drug discovery, this review reveals that while some branches of informatics are being utilized extensively, other methods have not been fully explored. By summarizing nine representative groups of informatics methods (see Figure 1 and Table 1), we highlight these disparities and, by extension, areas of opportunity for future research.

**Table 1:** Summary of popular computational drug discovery methods described in this review and their applicability to NP drug discovery, stratified by the major branches of informatics discussed in this review.

**Informatics branch**	**Method**	**Use with NPs**
Cheminformatics	QSAR analysis (section 3.1.1)	Multiple
	Molecular docking (section 3.1.2)	Multiple
	Computational library design (section 3.1.3)	Multiple
Bioinformatics	Gene expression perturbation (section 4.1.1)	Little to none
	Protein structure/function modeling (section 4.1.2)	Multiple
	Phylogenetic approaches (section 4.1.3)	Multiple
Semantic methods	Literature mining (section 5.1.1)	Limited
	EHR mining (section 5.1.2)	None
	Linking HTS data to effects (section 5.1.3)	Little to none

Pharmacologists and the pharmaceutical industry have championed the use of advanced cheminformatics techniques in concert with cutting-edge biotechnology innovations. Although NP drug discovery has always been a hallmark activity in pharmacology, pharmaceutical researchers have only applied these cheminformatic techniques to NPs rather recently. Both QSAR (section 3.1.1) and docking simulations (section 3.1.2) are standard practice for studying the therapeutic potential and mechanisms of NPs. There is also a fair number of NP library studies (section 3.1.3) that have been used to success—especially when focused on antibodies (Hoogenboom, 2005)—leading to the discovery of drugs, such as adalimumab (Jespers et al., 1994), ecallantide (Markland et al., 1996), and others (Nixon et al., 2014). As computing power improves, it is likely that we will see similar attention be paid to more challenging NP classes, such as venom peptides and other macromolecular compounds.

Bioinformatics demonstrates a similar trend, albeit somewhat earlier in its development (with regards to NP drug discovery) than cheminformatics. The bioinformatics methods covered in this review are intriguing in that each is a technique originally intended for uses other than drug discovery. Differential gene expression analysis (section 4.1.1) was originally used to explore differences between cell lines and disease states rather than the effects of drug perturbation, although the conceptual jump in applying expression analysis to drug discovery is arguably an obvious one. However, due to this technique's relatively recent emergence, few examples using NPs (as opposed to non-NP small molecule candidates) currently exist in the literature, none of which are truly data-driven (i.e., agnostic to both specific diseases and specific NP drug candidates). Nonetheless, analyses targeted toward specific diseases compared against the Connectivity Map dataset have resulted in two substantial discoveries based on plant metabolites: Celastrol as a treatment for acute myeloid leukemia (Hassane et al., 2008), and gedunin as a treatment for prostate cancer (Hieronymus et al., 2006). Therefore, the preliminary groundwork for truly data-driven drug discovery for NPs via perturbational differential expression analysis has already been established. For further examples of the successes of the Connectivity Map approach to data-driven drug discovery overall, we direct readers to a previous review by Musa et al. (2017). Phylogenetics (section 4.1.3)—one of the earlier uses for computers in biology—has become known for its diverse areas of application, including drug discovery. Since NPs come from organisms that can be studied in a phylogenetic context, bioinformaticians have realized just how valuable of a tool this can be for NP drug discovery, and a number of completed and ongoing research initiatives capitalize on this.

Semantic methods have been used much less frequently for drug discovery than the other branches of informatics, and even less so for NPs. Only a few sparse examples of literature mining applications (section 5.1.1) exist for NP drug discovery. A few studies show that ontologies and similar methods that link experimental evidence to HTS data and structured knowledge representations (section 5.1.3) could easily be adapted to perform preliminary validation for expensive and time-consuming manual experimentation to prove therapeutic activity in NPs, but the actual use of these methods for this purpose is also virtually non-existent. EHRs and other clinical data resources are in a similar situation—as far as we can tell, there are currently no published examples of clinical data mining (section 5.1.2) being used to discover therapeutic associations from NPs.

### 6.2. Data Needs for NP Drug Discovery

Throughout this review, we have touched upon computational and informatics methods with varying data needs, and have naturally mentioned several data resources that are dedicated to (or have strong relevance to) NP drug discovery. Just as certain discovery methods are enabled by characteristics specific to NPs, certain data types and dimensions are as well. This includes taxonomic/evolutionary data (Cordell, 2000; Larsen et al., 2005), primary (i.e., “intended”) targets and functions of NPs in nature (Bernardoni et al., 2014), the crude composition of NPs (often leading to synergistic effects, analogous to drug combination therapies) (Borkow et al., 1993; Casewell et al., 2013), and others specific to particular classes of NPs. A more comprehensive description of NP databases is presented in a review by Xie et al. (2015), but here we will cover some of them in brief as they pertaining to specific data needs.

The diversity and complexity of data types relevant for NP drug discovery research poses challenges in storing, representing, and exchanging these data. An immediate consequence is that many NP databases are limited to a narrow range of closely related NPs, which results in data fragmentation for the sake of completeness (Williams, 1997). ConoServer (Kaas et al., 2011) and ArachnoServer (Pineda et al., 2017) are two NP databases with rich and highly descriptive data, but each only applies to toxins produced by a single clade of species. One partial solution to this problem is to form dedicated efforts within larger, more general purpose databases that are dedicated to improving the representation of NPs, which is the approach taken by the Tox-Prot manual annotation program within UniProtKB/Swiss-Prot (Jungo et al., 2012). However, this does not completely resolve the greater issue of being able to leverage all important data types that are unique to certain classes of NPs. One other advantage that larger database efforts have over smaller, specialized NP databases is the presence of APIs and other tools that enable computational access. Many of the specialized databases do offer the ability to download data in bulk, but these can be incomplete and out-of-date. Furthermore, APIs can assist in making databases interoperable—an integrated network of specialized and well-annotated databases that can exchange semantic knowledge solves the issue of adequately representing granular characteristics while providing many of the benefits of larger data repositories.

Fragmentation of NP databases has also led to issues in maintaining those databases in the event of funding inconsistencies and institutional career changes—an issue that is at least partially safeguarded against when data resources are maintained by larger teams with more robust operating budgets. Three examples of now-defunct NP databases are the Traditional Chinese Medicine Systems Pharmacology (TCMSP) database (Ru et al., 2014), the Animal Toxin Database (ATDB) (He et al., 2007), and the SuperNatural database (Dunkel et al., 2006). Smaller NP databases can also suffer from issues like having unwieldy and non-descriptive URLs, such as that for the Tea Metabolome Database (found at http://pcsb.ahau.edu.cn:8080/TCDB/f) (Yue et al., 2014). Furthermore, if ownership of such a database changes, or if the principle investigator moves to a new institution, the URL would likely break, creating issues in finding the database when reading the manuscript that describes it—a phenomenon sometimes referred to as “link rot” (Markwell and Brooks, 2003).

Taking into account these and related issues, a wealth of opportunity is available for informatics researchers and data scientists to improve the quality, quantity, and interconnectedness of NP databases and knowledge representations. In the following section, we will reiterate these and other areas of importance for the near future, as elucidated over the course of this review.

### 6.3. A Road Map for the Future of Natural Product Drug Discovery

In spite of the disparities outlined above, renewed interest in bioontologies, semantic knowledge integration, and data-driven approaches to drug discovery suggests that this could be in the early stages of change. This review brings to light several concrete ways that the research industry could address existing issues and encourage the development of new innovations for NP drug discovery:

1. **Creating new ontology resources**: Structured semantic knowledge resources for NPs and NP drug discovery are scarce. Most databases are either overly general or overly specific, and therefore cannot capitalize on many of the unique characteristics demonstrated by entire classes of NPs. Resources with the appropriate ontological commitment are necessary to support the integration of the methods we have described—specifically, new standards compliant ontologies and tools for performing inference over (and between) these ontologies. To increase impact, these new ontologies should aim to span the translational divide, linking concepts that join fundamental biological characteristics of NPs to the clinically meaningful effects those NPs exert on the human body. Alternatively, the design of tools and frameworks that link more specialized ontologies (e.g., covering only taxonomy of NPs, or molecular targets of NPs) that together bridge this gap could be used to accomplish the same goal.
2. **Generating public HTS data for NPs**: Although the biomedical community is experiencing a deluge of multi-omic HTS data, the vast majority of non-human species are underrepresented or completely absent in public repositories. Unless more resources are devoted to publishing multi-omics data for species of interest to NP drug discovery, many of the discovery methods we have discussed will remain out-of-reach to most researchers.
3. **Utilizing clinical data**: New collaborative efforts such as OHDSI and eMERGE enable greater access to real clinical data that can be used for both discovery and evaluation of new drugs. As coverage of NPs improves in semantic knowledge resources, the ability to perform inference on NPs using observational data will improve as well.

## Author Contributions

JR and NT conceived of, wrote, and edited the content of this review.

### Conflict of Interest Statement

The authors declare that the research was conducted in the absence of any commercial or financial relationships that could be construed as a potential conflict of interest.
